# Value of FDG PET/CT in the assessment of patients with colon cancer comparing to stand-alone CT

**DOI:** 10.1186/1470-7330-14-S1-P29

**Published:** 2014-10-09

**Authors:** Kaveh Akbari, Franz Fellner, Klaus Emmanuel, Werner Langsteger, Mohsen Beheshti

**Affiliations:** 1 Department of Radiology, General Hospital Linz, Austria

## Purpose

To evaluate the potential of FDG PET/CT vs. stand-alone CT in the assessment of histopathologically verified colon cancer in primary staging, re-staging and follow up.

## Material and methods

70 patients (39 men, 31 women, mean age 70.7 ± 10.7 years) were included in this retrospective study: 28 (40%) primary staging, 28 (40%) re-staging and 14 (20%) follow-up patients.

Fifty-eight (58/70) patients (83%) had a primary tumour stage of ≥ T3. Patients with a known secondary carcinoma were excluded. Diagnostic contrast-enhanced CT was available in all patients (together with PET or in a separate setting with the same acquisition parameters).

The CT and FDG PET reports were examined for all patients. In discordant cases, images of both modalities were re-evaluated by a radiologist and a specialist in nuclear medicine separately.

All results were verified with histological findings or imaging and/or clinical follow-up studies for at least six months.

## Results

In the preoperative setting, additional FDG PET had an influence on the staging in 11 (11/28) patients (39%) comparing to CT alone:

Nine (9/28) patients (32%) were downstaged, 6 of them with suspicious organ metastases, 3 patients with suspicious lymph node metastases and 1 patient with both suspicious organ metastases and lymph nodes metastases on CT.

Two (2/28) patients (7%) were upstaged by FDG PET/CT, one of them with an unclear lung lesion on CT and a malignant hilar lymph node. The second patient showed peritoneal carcinosis on FDG PET.

Comparing with stand-alone CT, only 3 (3/42) patients (7%) from the restaging and follow-up group were downstaged by additional FDG PET, while concordant findings were seen on both imaging modalities for the rest of the patients.

## Conclusion

This study clearly showed that for primary staging of distant metastases in colon cancer patients FDG PET/CT is more advantageous and overcomes the lower specificity of CT alone.

Comparing both modalities in postoperative cases, FDG PET provides additional findings only in few cases.

